# Impact of the Covid-19 pandemic on perceptions and behaviors of university students in Vietnam

**DOI:** 10.1016/j.dib.2020.105880

**Published:** 2020-06-19

**Authors:** Duy Van Nguyen, Giang Hoang Pham, Dat Ngoc Nguyen

**Affiliations:** a Quantitative Analysis Center, 9/82 Chua Lang st, Dong Da dist, Hanoi, Vietnam; b Foreign Trade University, 91 Chua Lang st, Dong Da dist, Hanoi, Vietnam

**Keywords:** Covid-19, Coronavirus, Epidemic, Students, Perceptions and behaviors, Work and study, Travel and tourism, Decisions

## Abstract

This article presents a novel data set on perceptions and behaviors of university students collected after the beginning of the Covid-19 outbreak in Vietnam. Our questionnaire design is based on employing both qualitative interview with students and survey of SARS literature, probing into the sensitivity of students toward the crisis in making crucial decisions of daily routines, as well as future travel plans in presence of a grave health concern. The data set consists of 440 valid responses from Vietnamese university students through Internet platforms (Facebook, Google Form). Besides descriptive statistics, this article also includes the results of explanatory factor analysis, which may serve as a good reference for future studies.

**Specifications Table**

**Table:** 

**Subject**	Social Sciences (General)
**Specific subject area**	Epidemiology, Infectious Diseases, Student behaviours and perceptions, Econometric analysis
**Type of data**	Table
**How data were acquired**	Survey Questionnaire (included in Supplementary Materials)
**Data format**	Raw, analysed
**Parameters for data collection**	Respondents are randomly chosen for survey, but exclusively university students, including all in areas affected and not affected by the pandemic.
**Description of data collection**	The surveys were administered to university students between March 16 and March 22, with the support of Internet platforms (Facebook, Google Form), and resulted in valid 440 responses.
**Data source location**	Region: Asia Country: Vietnam
**Data accessibility**	Mendeley depository Direct URL: https://data.mendeley.com/datasets/c7s3pc6jy9/1

**Value of the data**

• The data can be used to uncover the impacts of the Covid-19 pandemic on perceptions and behaviors (work/study, move and travel) of Vietnamese university students in presence of public health crisis.
• The data is useful in providing further insights into the sensitivity of student consumers toward crisis in making their daily decisions and plans.
• The data serve well the purpose of providing guidance for tourism industry in formulation of recovery strategy in the post-crisis.
• The data is timely and valid with support of mixed methodology, and thus suitable to be used in across countries or dynamic analysis.

## 1 Data description

Since first emerging in Wuhan, China in December 2019, the disease caused by novel coronavirus SARS-CoV-2 has taken on pandemic proportions and severely affected the global public health and economy as well (WHO, 2020). As a country in close proximity to the initial epicenter of the outbreak, Vietnam faces a high risk of infection, and to combat the spread of virus all of the non-heath related activities were put on the brakes. Despite the validity of such responses in practice, they have widely influenced our way of living, but the degree of impact is not yet definite. With a focus on students as subject of investigation, this data set is aimed to enhance our knowledge repertoire regarding various aspects of the Covid-19 pandemic, in particular how student's behavior and perceptions may change due to the public health crisis. Our survey probes into the sensitivity of students toward crisis in making crucial decisions of daily routines, as well as future travel plans in presence of a grave health concern. In this sense, the data serve well the purpose of providing guidance for tourism industry in formulation of recovery strategy in the post-crisis.

The data collection was conducted between March 16 and March 22 after the beginning of the Covid-19 outbreak in Vietnam. Most infections in this period were imported cases when Vietnamese returned home from other countries, such as Malaysia, Britain, and France. At the end of the period, there were a total of 113 cases tested positive, of which just a handful were infected cases among community with untraceable source. Since 23 January when the first two cases were reported, Vietnam's government has taken proactive measures to prevent and contain the spread of the virus, from warnings against social gatherings and outdoor activities to strict quarantine for at-risk clusters. During the time of survey, there was not yet a uniformly applied measure until April 1 when the government ordered a nationwide isolation. With the support of Internet platforms (Facebook, Google Form), the survey resulted in valid 440 responses. Specifically, the questionnaire was structured in two parts: the first piece of information covered is demographic characteristics, including gender, household head's occupation, extra job status, monthly income, location; and the second part starts with general impacts felt during the outbreak, and then focuses on attitude toward and preferences for different decisions during and in the post-pandemic. Table 1 describes the characteristics of students in our sample. Table 2 summarizes the descriptive results of the responses to the questionnaire. The original data can be found in CSV format in the data repository.

**Table 1:** Respondents’ characteristics.

Variables	Categories				
Gender	Female (83.18%)	Male (16.82%)			
Household head's occupation	Officer (40.68%)	Farmer (21.59%)	Worker (3.86%)	Businessperson (26.36%)	Others (7.50%)
Extra job participation	Yes (57.50%)	No (42.50%)			
Monthly income (millions VND)	Lower than 5 (88.18%)	5–10 (9.55%)	10–15 (1.14%)	More than 15 (1.14%)	
Location	North (90.00%)	Central (8.86%)	South (1.14%)		

Note: Location is constructed based on the information of the city where the students are currently living, and categorized into 3 main groups: North, Central, South of Vietnam.

**Table 2:** Descriptive results of students’ responses of the survey.

No.	Items	*N*	Min	Max	Mean	S.D.
1	Covid-19 has greatly affected work/study activities	440	1	5	4.05	0.98
2	Covid-19 has greatly affected moving habits	440	1	5	3.66	1.07
3	Work/study activities are transformed during Covid-19 pandemic	440	1	5	4.44	0.91
4	Work/study loads are reduced	440	1	5	3.74	1.25
5	Covid-19 has greatly affected travel plans	440	1	5	4.23	1.00
6	You are concerned that going to work/university is not safe amid the pandemic	440	1	5	4.21	0.88
7	You feel that your time spent on working/studying is less productive	440	1	5	3.37	1.15
8	Going to crowded places during the outbreak is dangerous	440	1	5	4.30	0.97
9	Moving by public transportation during the pandemic is dangerous	440	1	5	4.38	0.93
10	You think of reducing duration of face-to-face meetings and teamwork activities	440	1	5	3.54	0.95
11	Avoiding going to places that have been affected by Covid-19 pandemic is necessary	440	1	5	4.49	0.81
12	You prefer tourist destinations in close proximity of your living area if travelling in the pandemic	440	1	5	3.58	1.26
13	You think of avoiding traveling in groups (shared meals and transportation) during Covid-19 pandemic	440	1	5	4.31	0.97
14	You prefer to travel with family and relatives during the pandemic	440	1	5	3.16	1.34
15	After the Covid-19 pandemic, going to crowded places is dangerous	440	1	5	3.21	1.11
16	After Covid-19, you are still worried about moving by public transportation	440	1	5	3.63	1.13
17	You will continue to reduce duration of face-to-face meetings and teamwork activities after Covid-19 pandemic	440	1	5	3.28	1.02
18	You will keep avoiding going to places that have been affected by Covid-19 pandemic	440	1	5	3.34	1.14
19	After Covid-19, you still prefer tourist destinations in close proximity of your living area	440	1	5	3.28	1.07
20	You think of avoiding traveling in groups (shared meals and transportation) after Covid-19 pandemic	440	1	5	3.25	1.15
21	After Covid-19, you prefer to travel with family and relatives	440	1	5	3.38	1.09

## 2 Experimental design, materials and methods

To construct the survey, we conducted a qualitative interview with randomly chosen 12 Vietnamese students, where open-ended questions were used for the exploratory purpose. Three main questions are (1) how have the Covid-19 outbreak generally affected your life? (2) what activities do you often participate in or avoid during the Covid-19 pandemic? (3) do you have any post-crisis plans, if yes what are they? Answers to these questions indicate the aspects of students’ life most likely influenced can be clustered around three groups of activities: work/study, move and travel. Perceptions of high risks of infection among community have brought about radical changes to their daily routines. For example, they had to get used to online lectures delivered on Internet platforms (e.g. Zoom, Microsoft Teams or Google's Hangout), and temporarily abandoned their move intentions and travel plans. Further, design of our survey was supplemented by the theoretical framework of perceived risk factors [1] and literature on the impacts of SARS on tourist consumption [2,3].

The survey instrument consisted of 26 items, including 21 statements of specific impact of the COVID-19 which require participants to rate on a 5-point Likert scale, particularly 1 = Totally disagree; 2 = Somewhat disagree; 3 = Neither agree nor disagree; 4 = Somewhat agree; 5 = Totally agree. We decided to employ online survey approach using the Internet platforms (Facebook, Google Form) with consent obtained from each respondent [4]. All of the survey items were obliged to be answered, thus no missing data was reported. In Table 3, we also provided the results of exploratory factor analysis with SPSS software, demonstrating that the 21 items were saliently loaded onto three dimensions, namely, perceptions of general impacts of the pandemic, attitude toward and preferences for specific decisions, which was further delineated into three sub-groups (1) AP1, AP3, AP4, AP6, AP8 named the move-related behaviours; (2) AP7, AP9 referred to as travel-related behaviours; (3) AP2, AP5 called work-related behaviours, and lastly the dimension reflecting after-pandemic decisions and plans. The analysis was grounded on relevant ratios such as Kaiser-Meyer-Olkin statistic (KMO) equal to or higher than 0.5, Barlett test with p-value smaller than 0.05, and average variance extracted over 50%, factor loadings of each item > 0.5 (Table 3).

**Table 3:** Exploratory factor analysis results.

	Code	Items	General impacts	Move	Travel	Work	After-pandemic general impacts
**General impacts**	GI3	Work/study activities are transformed during Covid-19 pandemic	.717				
	GI1	Covid-19 has greatly affected work/study activities	.675				
	GI5	Covid-19 has greatly affected travel plans	.688				
	GI4	Work/study loads are reduced	.609				
	GI2	Covid-19 has greatly affected moving habits	.546				
**During the pandemic**	AP4	Moving by public transportation during the pandemic is dangerous the Covid-19 pandemic is dangerous		.802			
	AP3	Going to crowded places during Covid-19 pandemic is dangerous		.772			
	AP1	You are concerned that going to work/university is not safe amid the pandemic		.747			
	AP6	Avoiding going to places that have been affected by Covid-19 pandemic is necessary		.725			
	AP8	You think of avoiding traveling in groups (shared meals and transportation) during Covid-19 pandemic		.649			
	AP7	You prefer tourist destinations in close proximity of your living area if travelling in the pandemic			.806		
	AP9	You prefer to travel with family and relatives during the pandemic			.747		
	AP2	You feel that your time spent on working/studying is less productive				.893	
	AP5	You think of reducing duration of face-to-face meetings and teamwork activities				.518	
**After Covid-19 pandemic**	AP6S	You will keep avoiding going to places that have been affected by Covid-19 pandemic					.800
	AP8S	You think of avoiding traveling in groups (shared meals and transportation) after Covid-19 pandemic					.797
	AP7S	After Covid-19, you still prefer tourist destinations in close proximity of your living area					.769
	AP3S	After Covid-19 pandemic, going to crowded places is dangerous					.731
	AP4S	After Covid-19, you are still worried about moving by public transportation					.678
	AP5S	You will continue to reduce duration of face-to-face meetings and teamwork activities after Covid-19 pandemic					.618
	AP9S	After Covid-19, you prefer to travel with family and relatives					.594

Note: Numerical values in the table are factor loadings corresponding to each item in the EFA.

## Declaration of Competing Interest

The authors declare that they have no known competing financial interests or personal relationships which have, or could be perceived to have, influenced the work reported in this article.

## Appendix Supplementary materials

Image, application 1

Image, application 2
