# Supplementary material for: Impact of the Covid-19 pandemic on perceptions and behaviors of university students in Vietnam
Source: Data Brief. 2020 Jun 19;31:105880. doi: 10.1016/j.dib.2020.105880 (PMC7303612; doi:10.1016/j.dib.2020.105880)
Supplement: Supplementary file 1 [file mmc1.pdf]

A survey on impacts of the Covid-19 pandemic on perceptions and behaviors of Vietnamese students

Dear Mr/Mrs,

We are a group of researchers and currently conducting a study on impacts of the Covid-19 pandemic on perceptions and behaviors of Vietnamese students. This study is aimed at objectively assessing how university students' activities have been affected due to the epidemic, and generally enhancing our knowledge repertoire of various aspects of public health crisis.

To achieve those objectives, we look forward to your support by answering the questions below. All of your ideas are useful to the research team, and none are true or false. Your personal information (if any) is kept confidential. If you have any questions about the research, please contact us via email: duynv@qaglobal.edu.vn!

I. Questionnaire

Please answer the questions below by circling your level of agreement corresponding to the given statements. In which 1 = Totally disagree; 2 = Somewhat disagree; 3 = Neither agree nor disagree; 4 = Somewhat agree; 5 = Totally agree

No.	Items	Level of agreement				
1	Covid-19 has greatly affected work/study activities	1	2	3	4	5
2	Covid-19 has greatly affected moving habits	1	2	3	4	5
3	Work/study activities are transformed during Covid-19 pandemic	1	2	3	4	5
4	Work/study loads are reduced	1	2	3	4	5
5	Covid-19 has greatly affected travel plans	1	2	3	4	5
6	You are concerned that going to work/university is not safe amid the pandemic	1	2	3	4	5
7	You feel that your time spent on working/studying is less productive	1	2	3	4	5
8	Going to crowded places during the outbreak is dangerous	1	2	3	4	5
9	Moving by public transportation during the pandemic is dangerous	1	2	3	4	5
10	You think of reducing duration of face-to-face meetings and teamwork activities	1	2	3	4	5
11	Avoiding going to places that have been affected by Covid-19 pandemic is necessary	1	2	3	4	5
12	You prefer tourist destinations in close proximity of your living area if travelling in the pandemic	1	2	3	4	5
13	You think of avoiding traveling in groups (shared meals and transportation) during Covid-19 pandemic	1	2	3	4	5
14	You prefer to travel with family and relatives during the pandemic	1	2	3	4	5

15	After the Covid-19 pandemic, going to crowded places is dangerous	1	2	3	4	5
16	After Covid-19, you are still worried about moving by public transportation	1	2	3	4	5
17	You will continue to reduce duration of face-to-face meetings and teamwork activities after Covid-19 pandemic	1	2	3	4	5
18	You will keep avoiding going to places that have been affected by Covid-19 pandemic	1	2	3	4	5
19	After Covid-19, you still prefer tourist destinations in close proximity of your living area	1	2	3	4	5
20	You think of avoiding traveling in groups (shared meals and transportation) after Covid-19 pandemic	1	2	3	4	5
21	After Covid-19, you prefer to travel with family and relatives	1	2	3	4	5

II. GENERAL INFORMATION

Please provide information by checking the corresponding box below:

1. Gender: ❒ Male ❒ Female
2. Your household head's occupation
   - ❒ Farmer ❒ Officer
   - ❒ Businessperson ❒ Worker
   - ❒ Others
3. Do you have an extra job?
   - ❒ Yes ❒ Không
4. What is your monthly income (in millions Vietnamdong)?
   - ❒ Lower than 5 ❒ From 5 to 10
   - ❒ From10 to 15 ❒ More than 15
5. Your location (Province): ………………………………………………….

If you would like to receive our research and survey results, please leave your email address here: ……………………………………………………………….

Thank you!
